# Clinical Impact of Pre-Procedural Percutaneous Coronary Intervention in Low- and Intermediate-Risk Transcatheter Aortic Valve Replacement Recipients

**DOI:** 10.3390/jpm11070633

**Published:** 2021-07-03

**Authors:** Max-Paul Winter, Thomas M. Hofbauer, Philipp E. Bartko, Christian Nitsche, Matthias Koschutnik, Andreas A. Kammerlander, Carolina Donà, Georg Spinka, Fabian Spinka, Martin Andreas, Markus Mach, Raphael Rosenhek, Irene M. Lang, Julia Mascherbauer, Christian Hengstenberg, Georg Goliasch

**Affiliations:** 1 Department of Cardiology, Internal Medicine II, Medical University of Vienna, Waehringer Guertel 18-20, 1090 Vienna, Austria; max-paul.winter@meduniwien.ac.at (M.-P.W.); thomas.hofbauer@meduniwien.ac.at (T.M.H.); philippemanuel.bartko@meduniwien.ac.at (P.E.B.); christian.nitsche@meduniwien.ac.at (C.N.); matthias.koschutnik@meduniwien.ac.at (M.K.); andreas.kammerlander@meduniwien.ac.at (A.A.K.); carolina.dona@meduniwien.ac.at (C.D.); georg.spinka@meduniwien.ac.at (G.S.); Fabian.spinka@gmail.com (F.S.); raphael.rosenhek@meduniwien.ac.at (R.R.); irene.lang@meduniwien.ac.at (I.M.L.); julia.mascherbauer@meduniwien.ac.at (J.M.); christian.hengstenberg@meduniwien.ac.at (C.H.); 2 Department of Cardiac Surgery, Medical University of Vienna, Waehringer Guertel 18-20, 1090 Vienna, Austria; martin.andreas@meduniwien.ac.at (M.A.); markus.mach@meduniwien.ac.at (M.M.)

**Keywords:** transcatheter aortic valve replacement, coronary artery disease, percutaneous coronary intervention, low-risk, intermediate-risk

## Abstract

The clinical relevance of as well as the optimal treatment strategy for coronary artery disease (CAD) in patients undergoing transcatheter aortic valve replacement (TAVR) for severe aortic stenosis (AS) are unclear. Current data are conflicting, and mainly derived from high-risk patients. We aimed to investigate the feasibility and safety of complete revascularization prior to TAVR for severe AS in low- and intermediate-risk patients. We enrolled 449 patients at low (STS score < 4%) and intermediate risk (STS score 4–8%) undergoing TAVR for severe AS and investigated the influence of recent (<3 months) and prior (>3 months) complete revascularization on clinical outcome. Primary study endpoint was all-cause mortality. Overall, 58% of patients had no or non-significant CAD; 18% had a history of complete revascularization prior to TAVR and 24% had complete revascularization shortly before TAVR. Two-year all-cause mortality was not different between patients with recent revascularization prior to TAVR and patients with no or non-significant CAD (13.7% vs. 14.2%, *p* = 0.905). Cox regression did not reveal an effect on all-cause mortality for recent revascularization. The present analysis reassures that percutaneous complete revascularization prior to TAVR procedures is neutral in terms of all-cause mortality in patients at low and intermediate surgical risk.

## 1. Introduction

Transcatheter aortic valve replacement (TAVR) has revolutionized therapy of patients with severe symptomatic aortic valve stenosis (AS) who are at prohibitive risk for conventional surgical aortic valve replacement (SAVR) [1]. A considerable increase in TAVR safety and efficacy, due to technological improvements and increasing operator experience, now expands TAVR as a valuable treatment option in patients with low to intermediate operative risk [2,3]. Considering that TAVR indication has expanded to include younger individuals with a longer life expectancy, attention now needs to be brought to a holistic treatment strategy, encompassing all relevant cardiac comorbidities [4].

The clinical relevance of coronary artery disease (CAD) in patients undergoing TAVR for severe aortic stenosis is unclear. Conflicting data results in uncertainty regarding the optimal treatment strategy in patients with concomitant CAD [5,6]. A recent meta-analysis including high-risk patients was unable to show benefit of revascularization prior to TAVR with respect to mortality [7]. Additionally, an observational study suggested that a high residual SYNTAX score (as defined as >14) is associated with an unfavorable cardiovascular outcome after 1 year [8]. 

Overall, data on pre-TAVR revascularization are not only conflicting but also primarily derived from high-risk patient populations, which does not reflect current clinical practice [9]. Thus, the aim of the present study was to evaluate (i) the feasibility and safety of conducting complete revascularization immediately pre-TAVR in this contemporary patient population, and (ii) to investigate the individual risk for prior or recent revascularization separated according to low or intermediate surgical risk. 

## 2. Materials and Methods

### 2.1. Study Population

In this observational study, we prospectively recruited consecutive patients (*n* = 449) undergoing TAVR for severe degenerative AS at a tertiary referral center between 16.11.2015 and 20.05.2020. Eligibility for, and decision to, perform TAVR was determined by a multidisciplinary board consisting of cardiac surgeons and interventional cardiologists. Recent percutaneous coronary intervention (PCI) was defined as complete revascularization within the preceding three months prior to TAVR. For revascularization, all patients were evaluated and discussed by a multidisciplinary team, and only patients deemed to benefit from revascularization were referred to PCI. Complete revascularization was defined according to consensus within the multidisciplinary board and based on a residual SYNTAX score < 8. Patients for whom coronary angiography data were available within six months of a scheduled TAVR did not undergo new angiography, if the previous examination did not reveal any coronary abnormalities (classified at the examiner’s discretion). Society of Thoracic Surgeons (STS) score was calculated as previously described [10]. Patients deemed at high perioperative risk as indicated by an STS score ≥8% were excluded from analyses. 

### 2.2. Clinical Measures and Follow Up

At study enrollment, medical history, current medication, electrocardiogram and echocardiography were routinely collected. Laboratory parameters were analyzed from venous blood samples according to the local laboratory’s standard procedure. Traditional cardiovascular risk factors were recorded according to the respective guidelines. Follow-up data were prospectively obtained from hospital readmission, assessment of outpatient records and contact with the patient and/or referring physician. Mortality was determined via retrieval query of the Austrian Death Registry. Record linkage revealed date of death and cause of death encoded according to the International Code of Diseases, Ver. 10 (ICD-10). All-cause mortality at two-year follow up was the primary endpoint [11] to compare patients with recent PCI to patients with no or non-significant CAD.

### 2.3. Statistical Methods

Normality of numerical data was assessed using the Kolmogorov–Smirnov test. Since no assessed parameter was normally distributed, data are exclusively presented as the median and interquartile range (IQR). Two groups were compared using Wilcoxon signed-rank or the Mann–Whitney test of paired or unpaired data. Three groups were compared using the Kruskal–Wallis test. Chi square analysis was employed to compare demographic parameters between groups. Kaplan–Meier analysis was employed to assess the time-dependent discriminative power of variables. Mortality assessment was performed using Cox regression. Only variables with a *p*-value of < 0.10 in a univariable analysis were subsequently used for multivariable computation. Calculations of univariable analyses are provided in Table S1. Hazard ratios (HR) and 95% confidence intervals (CI) are presented. Statistical analyses were performed using SPSS 25.0 (IBM). A *p*-value of < 0.05 was considered statistically significant. Figures were generated using GraphPad Prism 8. Censored values are not indicated.

## 3. Results

### 3.1. Patient Characteristics

Between 16 November 2015 and 20 May 2020, we screened a total of 605 patients with severe AS undergoing TAVR at our center. Inclusion flow chart is depicted in Figure S1. A total of 59 (10%) patients had an STS score of ≥8% and were thus excluded from subsequent analysis. Another subset of patients with non-recent revascularization (*n* = 97, 16%) were also excluded. In total, 449 patients undergoing TAVR for severe AS were enrolled in the present study. Median age was 81 [IQR 77–85] years and 232 (52%) patients were male. STS score among all patients was 3.00 [2.12–4.23], with 313 (70%) patients at low surgical risk, and 136 (30%) at intermediate risk.

Aortic valve maximum velocity (Vmax) was 4.3 [IQR 4.0–4.8] m/sec; mean pressure gradient (mPG) was 46 [IQR 40–56] mmHg, and aortic valve area was 0.7 [IQR 0.6–0.8] cm^2^. TAVR was performed using a self-expandable device in 292 (65%), and with a balloon-expandable device in 157 (35%) patients. Detailed patient characteristics are given in Table 1.

### 3.2. Prevalence of CAD and Revascularization in Low- and Intermediate-Risk TAVR Patients

Overall, 58% had no or non-significant CAD; 18% a history of complete revascularization prior to valve implantation (> 3 months before TAVR) and 24% complete revascularization shortly before TAVR. Of those, 10 (8%) received PCI of the left main, 66 (50%) of the left artery descending (LAD), 32 (24%) of the circumflex (CX) and 51 (39%) of the right coronary artery (RCA).

STS score was not different between patients with recent revascularization vs. no or non-significant CAD (3.10 [2.20, 4.47] vs. 2.95 [2.06, 4.11], *p* = 0.1261), while EuroSCORE II was slightly lower in patients with recent revascularization (3.96 [2.66–4.97] vs. no or non-significant CAD 4.22 [3.63–5.29], *p* = 0.0278).

Patients with recent revascularization were more often male as compared to patients with no or non-significant CAD (63% vs. 47%, *p* = 0.0029). Patients undergoing recent revascularization showed a more pronounced cardiovascular risk phenotype as depicted by a higher prevalence of hyperlipidemia (84% vs. 611%, *p* < 0.0001), previous myocardial infarction (22% vs. 3%, *p* < 0.0001) and previous coronary artery bypass grafting (8% vs. 0%, *p* < 0.0001). Prevalence of diabetes mellitus (32% vs. 25%, *p* = 0.1547) and arterial hypertension (89% vs. 89%, *p* = 0.8461) did not differ between the two groups.

### 3.3. Clinical Outcome

**Figure 1:**
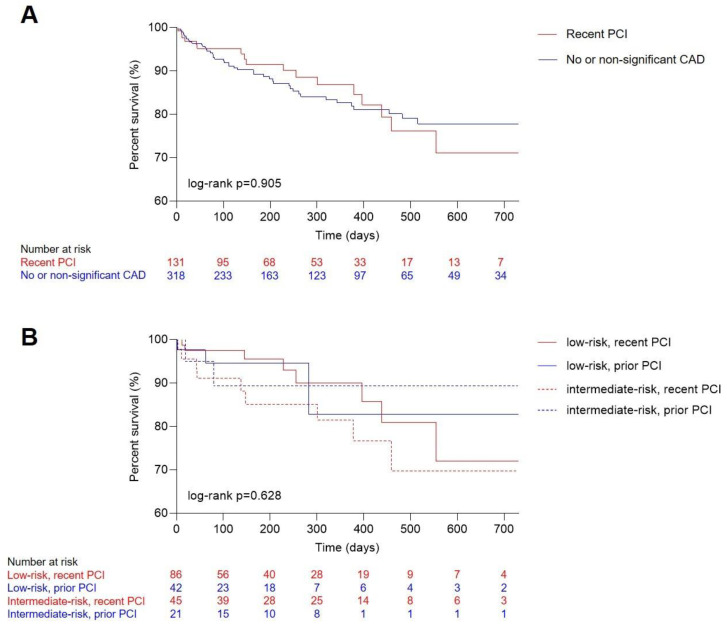
Outcome analysis for TAVR patients. Kaplan–Meier estimates of long-term mortality comparing patients with (**A**) no and non-significant CAD and recent complete revascularization (overall log-rank *p* = 0.905) and (**B**) patients with prior or recent full revascularization according to low and intermediate surgical risk (overall log-rank *p* = 0.628). CAD, coronary artery disease; PCI, percutaneous coronary intervention; TAVR, transcatheter aortic valve replacement.

Median follow up was 218 [IQR 84–422] days, with a median survival after TAVR of 613 [IQR 587–639] days. Overall, 63 (14%) patients died in the observational period. Death occurred in 18 (14%) patients with recent revascularization and 45 (14%) patients with no or non-significant CAD. Between these two groups, Kaplan–Meier analysis illustrated no difference (Figure 1A, log-rank *p* = 0.905). Further stratifying both groups into either low or intermediate risk also did not reveal differences in survival (Figure 1B, log-rank *p* = 0.628).

Using Cox regression analysis (Table 2), recent revascularization was not associated with long-term, all-cause mortality, with a crude HR of 1.082 ([95% CI 0.635–1.846], *p* = 0.771). This finding remained virtually unchanged even after adjusting for established cardiovascular risk factors, with an adjusted HR of 1.201 ([95% CI 0.683–2.111], *p* = 0.525). Similarly, stratifying patients based on low or intermediate risk, recent revascularization did not reveal an impact on mortality (Table 3). In low-risk patients, adjusted HR was 1.260 ([95% CI 0.551–2.880], *p* = 0.584). For intermediate-risk patients, adjusted HR was 1.363 ([95% CI 0.603–3.083], *p* = 0.457).

## 4. Discussion

Based on this large-scale outcome study, we identified that CAD is still one of the most frequent comorbidities in low- and intermediate-risk patients scheduled for TAVR. Furthermore, we could prove that PCI prior to TAVR is not associated with adverse long-term outcome regardless of calculated operative risk classification (low vs. intermediate peri-operative risk)

Our findings have important implications for contemporary shared decision making in the Heart Team as they reassure safety of percutaneous coronary and valvular interventions in low–intermediate risk patients.

Within the last decade, TAVR has become the standard of care for patients with symptomatic severe aortic stenosis in patients at high surgical risk. However, ongoing technical evolution of available TAVR prosthesis in conjunction with growing operator experience and novel possibilities of cardiac imaging has markedly decreased peri-interventional risk of TAVR procedures and now offers the possibility to expand these valuable treatment option to younger patients with lower risk. To date, data from two randomized trials (PARTNER 3, Evolut Low Risk) reported encouraging results with at least non-inferiority as compared to conventional aortic valve replacement [12,13]. Recently, the PARTNER 3 trial even reassured both the beneficial clinical outcome and the durability of TAVR bioprostheses in the long term follow up [12].

These findings have now led to the adaption of recent guidelines of the American College of Cardiology and American Heart Association with the introduction of this treatment option also in low–intermediate risk patients [2]. However, this expansion of TAVR as a treatment option in younger individuals, with longer life expectancy, now demands that attention is brought to a holistic treatment strategy, encompassing all relevant cardiac comorbidities. The practice of complete revascularization at the time point of valve replacement emanates not from randomized-controlled data, but relies on the clinical advantage of treating the obstructive disease when open heart surgery is considered [7]. With the expected increase in TAVR procedures, it is now crucial to reevaluate common practice and to devise consistent strategies with regard to the management of CAD based on solid outcome data. Therefore, we investigated the influence of recent complete revascularization in low- and intermediate-risk patients. In high-risk patients, it has been shown that significant CAD harbors no mortality risk in patients undergoing TAVR [14]. However, applicability of these observations are limited, as follow-up periods are comparatively short. Furthermore, especially when TAVR is performed in lower-risk patients, life expectancy is significantly longer, and deterioration of CAD is more likely. In the present analysis, we found indistinguishable clinical outcome at two-year follow up after TAVR in low- and intermediate-risk patients with complete revascularization immediately prior to TAVR as compared to patients with no or only non-significant CAD. Therefore, in cases that are deemed to benefit from complete revascularization after Heart Team evaluation, PCI before TAVR appears to be a safe and feasible strategy, translating into generally favorable post-interventional outcome. In the absence of contraindications, TAVR is increasingly considered in patients aged 65 to 80 years, where SAVR was treatment of choice due to durability of surgical valves [2]. In our study, median age was 81 years. Our patient cohort thus comprises this critical age range of 65 to years to a significant proportion. Our observations hereby support recent guidelines, and serve as an important information basis for shared decision making in the interdisciplinary Heart Team.

We observed a high prevalence of hyperlipidemia in patients undergoing recent revascularization compared to patients with no or non-significant CAD. Additionally, patients were more frequently male in the recent revascularization group. Diabetes mellitus tended to be more frequent in the former group, albeit not reaching statistical significance. Given that hyperlipidemia, diabetes and male sex are associated with cardiovascular events [15], revascularization might still be beneficial, improving long-term outcome beyond our timeframe of observation.

Robust data from randomized trials on whether PCI prior to TAVR results in improved outcome are lacking. Currently, the ACTIVATION trial (ISRCTN75836930) randomizes patients to PCI or no PCI prior to TAVR [16]. Similarly, the TAVI-PCI trial compares PCI before and post-TAVI (NCT04310046). In the NOTION-3 trial (NCT03058627) patients are randomized to TAVR only or TAVR with prior FFR-guided revascularization.

## 5. Limitations

Our study has several limitations. CAD was based on quantitative coronary angiography instead of hemodynamic assessment with fractional flow reserve or instantaneous wave-free ratio. Completeness of revascularization was defined at the operator’s discretion. Primary outcome of all-cause mortality was robust, as data were obtained from a systematic and centralized database. However, the observational nature of the present study may lead to confounding of results by hidden biases and unmeasured differences (like adherence to medical therapy) that are associated with clinical outcome. Other safety endpoints apart from mortality (like catheter-associated complications not resulting in death) were not investigated in this study.

## 6. Conclusions

Taken together, our data indicate that complete revascularization prior to TAVR implantation is a safe and feasible procedure in intermediate- and low-risk patients. Therefore, the presence of significant CAD in this patient cohort is not a reasonable comorbidity that indicates a potential benefit for conventional SAVR. For this purpose, it remains paramount to discuss the coronary anatomy in interdisciplinary Heart Teams, with shared decision making to identify those patients that truly benefit from complete revascularization.

## 7. Impact on Daily Practice

CAD is prevalent among patients with AS subjected to TAVR, even in patients deemed at low or intermediate surgical risk; however, whether previous PCI influences outcome remains uncertain. We found that revascularization prior to TAVR is not associated with outcome, irrespective of low or intermediate surgical risk as calculated by the STS score. While PCI is safe in AS patients at low- and intermediate-risk patients undergoing TAVR, interdisciplinary discussion regarding optimal timing of coronary revascularization should be emphasized.

## Figures and Tables

**Table 1:** Baseline characteristics of TAVR patients.

	All Patients	Recent Revascularization	No or Non-Significant CAD	*p* Value
Patients, *n* (%)	449 (100.00)	131 (29.18)	318 (70.82)	0.1261
STS score, median [IQR]	3.00 [2.12, 4.23]	3.10 [2.20, 4.47]	2.95 [2.06, 4.11]	0.2293
Low risk (<4%)	313 (69.71)	86 (65.65)	227 (71.38)	0.2293
Intermediate risk (4–8%)	136 (30.29)	45 (34.35)	91 (28.62)	1.000
EuroSCORE II, median [IQR]	4.17 [3.28, 5.13]	3.96 [2.66, 4.97]	4.22 [3.63, 5.29]	0.0278
Death at follow up, *n* (%)	63 (14.03)	18 (13.74)	45 (14.15)	
Age in years, median [IQR]	81 [77, 85]	80 [77, 85]	81 [77, 85]	0.2329
Male sex, *n* (%)	232 (51.67)	82 (62.60)	150 (47.17)	0.0029
BMI, median [IQR]	26.84 [23.53, 30.09]	27.37 [23.86, 30.20]	26.79 [23.46, 30.07]	0.4399
CAD, *n* (%)	226 (50.33)	131 (100.00)	95 (29.87)	<0.0001
Diabetes, *n* (%)	123 (27.39)	42 (32.06)	81 (25.47)	0.1547
Arterial hypertension, *n* (%)	399 (88.86)	117 (89.31)	282 (88.68)	0.8461
Hyperlipidemia, *n* (%)	304 (67.71)	110 (83.97)	194 (61.01)	<0.0001
Active smoker, *n* (%)	24 (5.35)	11 (8.40)	13 (4.09)	0.0650
Atrial fibrillation, *n* (%)	164 (36.53)	47 (35.88)	117 (36.79)	0.8548
Stroke, *n* (%)	31 (6.90)	6 (4.58)	25 (7.86)	0.2125
Peripheral arterial disease, *n* (%)	39 (8.69)	15 (11.45)	24 (7.55)	0.1819
Cerebral arterial disease, *n* (%)	69 (15.37)	26 (19.85)	43 (13.52)	0.0911
Liver disease, *n* (%)	21 (4.68)	5 (3.82)	16 (5.03)	0.5794
COPD, *n* (%)	52 (11.58)	12 (9.16)	40 (12.58)	0.3035
Previous MI, *n* (%)	39 (8.69)	29 (22.14)	10 (3.14)	<0.0001
Previous CABG, *n* (%)	11 (2.45)	11 (8.40)	0 (0.00)	<0.0001
Previous PCI, *n* (%)	131 (29.18)	131 (100)	0 (0.00)	<0.0001
Previous valve surgery, *n* (%)	30 (6.68)	3 (2.29)	27 (8.49)	0.0168
Pacemaker prior to TAVR, *n* (%)	49 (10.91)	17 (12.98)	32 (10.06)	0.3680
History of syncope, *n* (%)	64 (14.25)	17 (12.98)	47 (14.78)	0.6194
Recent revascularization, *n* (%)	131 (29.18)	131 (100.00)	0 (100.00)	<0.0001
Dyspnea at presentation (NYHA)				0.6069
NYHA I	30 (6.68)	9 (6.87)	21 (6.60)	
NYHA II	144 (32.07)	47 (35.88)	97 (30.50)	
NYHA III	254 (56.57)	67 (51.15)	187 (58.81)	
NYHA IV	21 (4.68)	8 (6.10)	13 (4.09)	
Aortic valve stenosis				
Vmax, m/sec, median [IQR]	4.3 [4.0, 4.8]	4.3 [4.0, 4.7]	4.3 [4.0, 4.8]	0.5681
mPG, mmHg, median [IQR]	46 [40, 56]	45 [40, 54]	47 [40, 56]	0.4579
AVA, cm^2^, median [IQR]	0.7 [0.6, 0.8]	0.7 [0.6, 0.8]	0.7 [0.6, 0.8]	0.7267
TAVR technique				
Self-expandable, *n* (%)	292 (65.03)	82 (62.60)	210 (66.04)	0.4869
Balloon-expandable, *n* (%)	157 (34.97)	49 (37.40)	108 (33.96)	0.4869
Echocardiographic parameters				
LV function				0. 8649
Normal, *n* (%)	291 (64.81)	83 (70.94)	208 (65.41)	
Mildly reduced, *n* (%)	39 (8.69)	11 (9.40)	28 (8.81)	
Moderately reduced, *n* (%)	41 (9.13)	14 (11.97)	27 (8.49)	
Severely reduced, *n* (%)	35 (7.80)	9 (7.69)	26 (8.18)	
IVS, mm, median [IQR]	15 [13, 17]	15 [14, 17]	15 [13, 17]	0.2865
Mitral regurgitation				0.5575
None, *n* (%)	100 (22.27)	27 (22.88)	73 (22.96)	
Mild, *n* (%)	143 (31.85)	48 (40.68)	95 (29.87)	
Moderate, *n* (%)	128 (28.51)	36 (30.51)	92 (28.93)	
Severe, *n* (%)	35 (7.80)	7 (5.93)	28 (8.81)	
Tricuspid regurgitation				0.0638
None, *n* (%)	167 (37.19)	58 (48.74)	109 (34.28)	
Mild, *n* (%)	114 (25.39)	29 (24.37)	85 (26.73)	
Moderate, *n* (%)	92 (20.49)	23 (19.33)	69 (21.70)	
Severe, *n* (%)	35 (7.80)	9 (7.56)	26 (8.18)	
Background medication				
ACE-I, *n* (%)	152 (33.85)	56 (42.75)	96 (30.19)	0.0106
ARB, *n* (%)	155 (34.52)	43 (32.82)	112 (35.22)	0.6274
Beta-blocker, *n* (%)	255 (56.79)	78 (59.54)	177 (55.66)	0.4504
Laboratory values				
eGFR, median [IQR]	50.0 [37.9, 63.3]	53.1 [40.6, 64.9]	48.2 [36.6, 63.2]	
Hematocrit, %, median [IQR]	35.3 [31.9, 39.1]	34.5 [30.8, 38.4]	35.7 [32.4, 39.3]	
proBNP, pg/mL, median [IQR]	1538 [646, 4000]	1362 [640, 3750]	1612 [647, 4141]	

Numerical data are given as the median [IQR] or percent. ACE-I, angiotensin-converting enzyme inhibitor; ARB, angiotensin receptor blocker; AVA, aortic valve area; BMI, body mass index; CABG, coronary arterial bypass graft; CAD, coronary artery disease; COPD, chronic obstructive pulmonary disease; CX, circumflex artery; eGFR, estimated glomerular filtration rate (measured as mL/min/1.73 m^2^); Hk, hematocrit; IQR, interquartile range; IVS, interventricular septum; LAD, left anterior descending artery; LM, left main; LV, left ventricle; MI, myocardial infarction; mPG, mean pressure gradient; NT-proBNP, N-terminal pro B-type natriuretic peptide; NYHA, New York Heart Association; PCI, percutaneous coronary intervention; RCA, right coronary artery; STS, Society of Thoracic Surgeons; sysPAP, systolic pulmonary artery pressure; TAVR, transcatheter aortic valve replacement; Vmax, maximum aortic valve velocity. Strata for LV function were defined as normal (LV ejection fraction [EF] > 50%), mildly reduced (EF 40–49%), moderately reduced (EF 30–39%) and severely reduced (EF < 30%).

**Table 2:** Influence of recent revascularization on all-cause mortality in TAVR patients after two-year follow up.

Factor	Adjusted HR	95% CI	*p*-Value
Male sex	1.533	0.904–2.602	0.113
Diabetes mellitus	2.314	1.381–3.879	0.001
BMI	0.924	0.866–0.986	0.018
Hyperlipidemia	0.593	0.355–0.991	0.046
eGFR	0.991	0.977–1.006	0.227
NT-proBNP per SD	1.114	0.898–1.382	0.326
Hematocrit	0.967	0.928–1.008	0.114
Recent revascularization	1.201	0.683–2.111	0.525

Multivariable Cox regression was used to assess the influence of recent revascularization on all-cause mortality in patients undergoing TAVR, after a median follow up of 218 [84, 422] days. BMI, body mass index; CI, confidence interval; eGFR, estimated glomerular filtration rate; HR, hazard ratio; NT-proBNP, N-terminal B-type natriuretic peptide; SD, standard deviation; TAVR, transcatheter aortic valve replacement.

**Table 3:** Influence of recent revascularization on all-cause mortality, stratified by low or intermediate risk.

Factor	Adjusted HR	95% CI	*p*-Value
**Low risk**			
Male sex	1.644	0.718–3.766	0.239
Diabetes mellitus	2.048	0.982–4.269	0.056
BMI	0.959	0.873–1.054	0.385
Hyperlipidemia	0.469	0.231–0.950	0.036
eGFR	0.985	0.965–1.005	0.134
NT-proBNP per SD	1.280	1.012–1.619	0.040
Hematocrit	0.974	0.921–1.029	0.346
Recent revascularization	1.260	0.551–2.880	0.584
**Intermediate risk**			
Male sex	2.048	0.942–4.450	0.070
Diabetes mellitus	2.711	1.208–6.085	0.016
BMI	0.894	0.810–0.986	0.025
Hyperlipidemia	0.598	0.261–1.370	0.224
eGFR	1.003	0.974–1.032	0.861
NT-proBNP per SD	0.703	0.410–1.206	0.201
Hematocrit	0.938	0.884–0.995	0.032
Recent revascularization	1.363	0.603–3.083	0.457

Multivariable Cox regression was used to assess the influence of recent revascularization on all-cause mortality in patients undergoing TAVR, after a median follow up of 218 [84, 422] days, stratified by low risk (STS score < 4%) and intermediate risk (STS score 4–8%). BMI, body mass index; CI, confidence interval; eGFR, estimated glomerular filtration rate; HR, hazard ratio; NT-proBNP, N-terminal B-type natriuretic peptide; SD, standard deviation; TAVR, transcatheter aortic valve replacement.

## Data Availability

Data presented in this study are available upon request from the corresponding author.

## Supplementary Materials

The following are available online at https://www.mdpi.com/article/10.3390/jpm11070633/s1, Figure S1: Flowchart of patients included into the study; Table S1: Univariable regression analysis for clinical variables.

## References

[1] Ielasi A., Latib A., Tespili M., Donatelli F. (2019). Current results and remaining challenges of trans-catheter aortic valve replacement expansion in intermediate and low risk patients. Int. J. Cardiol. Heart Vasc..

[2] Otto C.M., Nishimura R.A., Bonow R.O., Carabello B.A., Erwin J.P., Gentile F., Jneid H., Krieger E.V., Mack M., McLeod C. (2021). 2020 ACC/AHA Guideline for the Management of Patients with Valvular Heart Disease: Executive Summary: A Report of the American College of Cardiology/American Heart Association Joint Committee on Clinical Practice Guidelines. Circulation.

[3] Voigtlander L., Seiffert M. (2018). Expanding TAVI to Low and Intermediate Risk Patients. Front. Cardiovasc. Med..

[4] Winter M.P., Bartko P., Hofer F., Zbiral M., Burger A., Ghanim B., Kastner J., Lang I.M., Mascherbauer J., Hengstenberg C. (2020). Evolution of outcome and complications in TAVR: A meta-analysis of observational and randomized studies. Sci. Rep..

[5] Faroux L., Guimaraes L., Wintzer-Wehekind J., Junquera L., Ferreira-Neto A.N., Del Val D., Muntane-Carol G., Mohammadi S., Paradis J.M., Rodes-Cabau J. (2019). Coronary Artery Disease and Transcatheter Aortic Valve Replacement: JACC State-of-the-Art Review. J. Am. Coll. Cardiol..

[6] Nishimura R.A., O’Gara P.T., Bavaria J.E., Brindis R.G., Carroll J.D., Kavinsky C.J., Lindman B.R., Linderbaum J.A., Little S.H., Mack M.J. (2019). 2019 AATS/ACC/ASE/SCAI/STS Expert Consensus Systems of Care Document: A Proposal to Optimize Care for Patients with Valvular Heart Disease: A Joint Report of the American Association for Thoracic Surgery, American College of Cardiology, American Society of Echocardiography, Society for Cardiovascular Angiography and Interventions, and Society of Thoracic Surgeons. J. Am. Coll. Cardiol..

[7] Lateef N., Khan M.S., Deo S.V., Yamani N., Riaz H., Virk H.U.H., Khan S.U., Hedrick D.P., Kanaan A., Reed G.W. (2019). Meta-Analysis Comparing Outcomes in Patients Undergoing Transcatheter Aortic Valve Implantation with Versus Without Percutaneous Coronary Intervention. Am. J. Cardiol..

[8] Stefanini G.G., Stortecky S., Cao D., Rat-Wirtzler J., O’Sullivan C.J., Gloekler S., Buellesfeld L., Khattab A.A., Nietlispach F., Pilgrim T. (2014). Coronary artery disease severity and aortic stenosis: Clinical outcomes according to SYNTAX score in patients undergoing transcatheter aortic valve implantation. Eur. heart J..

[9] Waksman R., Rogers T., Torguson R., Gordon P., Ehsan A., Wilson S.R., Goncalves J., Levitt R., Hahn C., Parikh P. (2018). Transcatheter Aortic Valve Replacement in Low-Risk Patients with Symptomatic Severe Aortic Stenosis. J. Am. Coll. Cardiol..

[10] Barbash I.M., Finkelstein A., Barsheshet A., Segev A., Steinvil A., Assali A., Ben Gal Y., Vaknin Assa H., Fefer P., Sagie A. (2015). Outcomes of Patients at Estimated Low, Intermediate, and High Risk Undergoing Transcatheter Aortic Valve Implantation for Aortic Stenosis. Am. J. Cardiol..

[11] Bartko P.E., Arfsten H., Heitzinger G., Pavo N., Spinka G., Kastl S., Prausmuller S., Strunk G., Mascherbauer J., Hengstenberg C. (2019). Global regurgitant volume: Approaching the critical mass in valvular-driven heart failure. Eur. Heart J. Cardiovasc. Imaging.

[12] Mack M.J., Leon M.B., Thourani V.H., Makkar R., Kodali S.K., Russo M., Kapadia S.R., Malaisrie S.C., Cohen D.J., Pibarot P. (2019). Transcatheter Aortic-Valve Replacement with a Balloon-Expandable Valve in Low-Risk Patients. N. Engl. J. Med..

[13] Popma J.J., Deeb G.M., Yakubov S.J., Mumtaz M., Gada H., O’Hair D., Bajwa T., Heiser J.C., Merhi W., Kleiman N.S. (2019). Transcatheter Aortic-Valve Replacement with a Self-Expanding Valve in Low-Risk Patients. N. Engl. J. Med..

[14] Snow T.M., Ludman P., Banya W., DeBelder M., MacCarthy P.M., Davies S.W., Di Mario C., Moat N.E. (2015). Management of concomitant coronary artery disease in patients undergoing transcatheter aortic valve implantation: The United Kingdom TAVI Registry. Int. J. Cardiol..

[15] Mach F., Baigent C., Catapano A.L., Koskinas K.C., Casula M., Badimon L., Chapman M.J., De Backer G.G., Delgado V., Ference B.A. (2020). 2019 ESC/EAS Guidelines for the management of dyslipidaemias: Lipid modification to reduce cardiovascular risk. Eur. Heart J..

[16] Khawaja M.Z., Wang D., Pocock S., Redwood S.R., Thomas M.R. (2014). The percutaneous coronary intervention prior to transcatheter aortic valve implantation (ACTIVATION) trial: Study protocol for a randomized controlled trial. Trials.

