# Supplementary material for: Clinical Impact of Pre-Procedural Percutaneous Coronary Intervention in Low- and Intermediate-Risk Transcatheter Aortic Valve Replacement Recipients
Source: J Pers Med. 2021 Jul 3;11(7):633. doi: 10.3390/jpm11070633 (PMC8304453; doi:10.3390/jpm11070633)

Table S1 – univariable regression analysis for clinical variables

Variable	HR	95% CI	p-value
**Age**	1.014	0.980 – 1.049	0.422
**Male sex**	1.531	0.950 – 2.468	**0.080**
**BMI**	0.915	0.868 – 0.966	**0.001**
**CAD**	0.989	0.621 – 1.575	0.963
**Diabetes mellitus**	1.747	1.089 – 2.802	**0.021**
**Arterial hypertension**	0.678	0.348 – 1.322	0.254
**Hyperlipidemia**	0.646	0.402 – 1.038	**0.071**
**Active smoker**	0.956	0.347 – 2.631	0.931
**Atrial fibrillation**	1.170	0.731 – 1.875	0.513
**Stroke**	0.329	0.081 – 1.344	0.122
**PAD**	1.105	0.549 – 2.223	0.780
**Cerebrovascular AD**	0.760	0.400 – 1.447	0.404
**Liver disease**	1.383	0.504 – 3.792	0.529
**COPD**	0.925	0.443 – 1.929	0.835
**Previous MI**	0.844	0.405 – 1.760	0.651
**Previous CABG**	1.185	0.568 – 2.473	0.651
**Previous PCI**	0.952	0.579 – 1.564	0.846
**Previous valve surgery**	0.978	0.394 – 2.428	0.961
**PM prior to TAVR**	0.914	0.438 – 1.906	0.810
**History of syncope**	1.070	0.563 – 2.035	0.836
**Recent revascularization**	1.082	0.635 – 1.846	0.771
**eGFR**	0.979	0.968 – 0.991	**0.001**
**Hematocrit**	0.955	0.918 – 0.993	**0.021**
**proBNP per SD**	1.310	1.104 – 1.554	**0.002**
**LVEF**	1.103	0.884 – 1.377	0.386

Univariable Cox regression was used to assess the influence of established cardiovascular risk factors and recent revascularization on all-cause mortality in patients undergoing TAVR, after a median follow-up of 218 [84, 422] days. BMI body mass index, CABG coronary artery bypass graft, CAD coronary artery disease, CI confidence interval, COPD chronic obstructive pulmonary disease, eGFR estimated glomerular filtration rate, HR hazard ratio, LVEF left ventricular ejection fraction, MI myocardial infarction, proBNP N-terminal B-type natriuretic peptide, PAD peripheral artery disease, PCI percutaneous coronary intervention, PM pacemaker, SD standard deviation, TAVR transcatheter aortic valve replacement.

Figure S1 – flowchart of patients included into the study

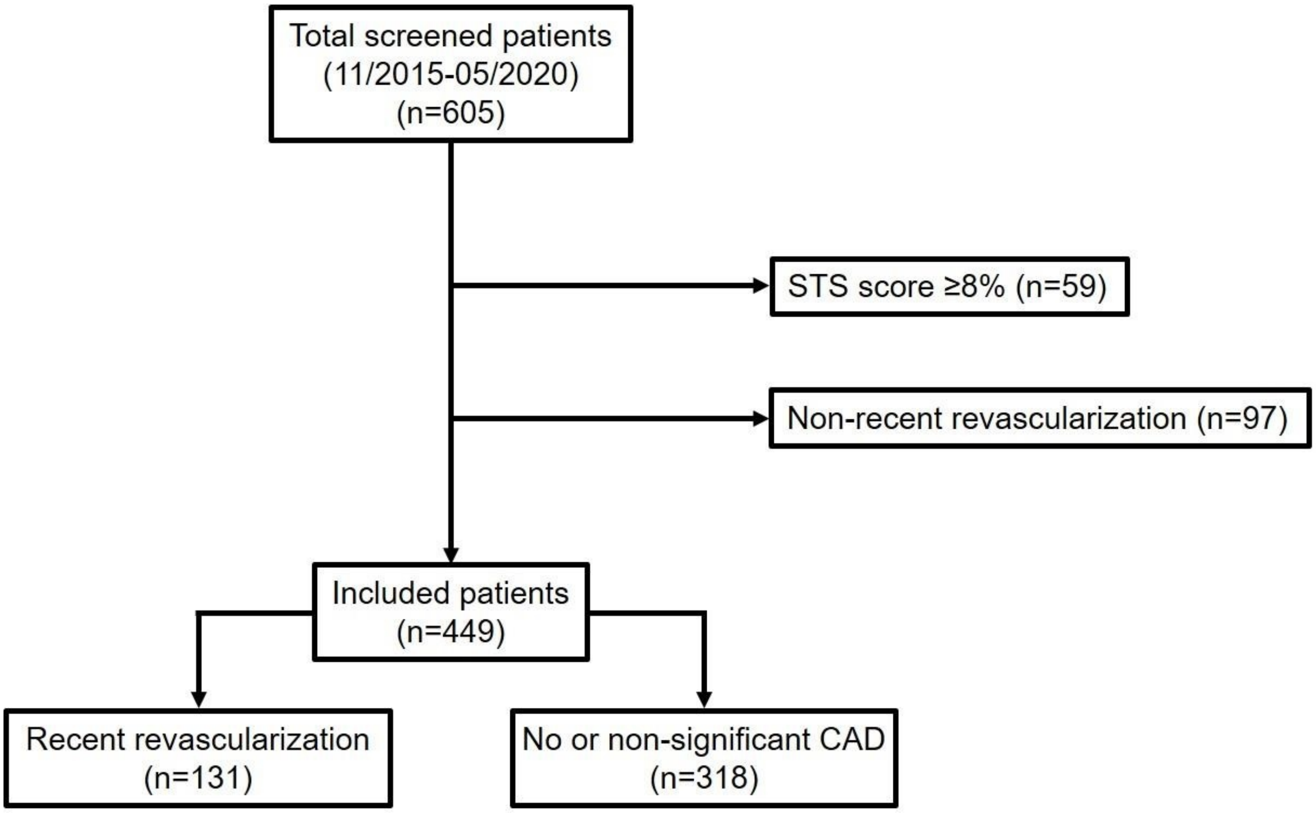

Supplement: Supplementary file 1 [file jpm-11-00633-s001.zip › jpm-1256883-SI.pdf]
